# Schemes for Single Electron Transistor Based on Double Quantum Dot Islands Utilizing a Graphene Nanoscroll, Carbon Nanotube and Fullerene

**DOI:** 10.3390/molecules27010301

**Published:** 2022-01-04

**Authors:** Vahideh Khademhosseini, Daryoosh Dideban, Mohammad Taghi Ahmadi, Hadi Heidari

**Affiliations:** 1 Institute of Nanoscience and Nanotechnology, University of Kashan, Kashan 8731753153, Iran; v_khademhosseini@grad.kashanu.ac.ir; 2 Department of Electrical and Computer Engineering, University of Kashan, Kashan 8731753153, Iran; 3 Nanotechnology Research Center, Nano-Physic Group, Department of Physics, Urmia University, Urmia 5756151818, Iran; mt.ahmadi@urmia.ac.ir; 4 James Watt School of Engineering, University of Glasgow, Glasgow G12 8QQ, UK; hadi.heidari@glasgow.ac.uk

**Keywords:** carbon nanotube (CNT), fullerene, graphene nanoscroll (GNS), multiple quantum dot islands, single electron transistor

## Abstract

The single electron transistor (SET) is a nanoscale switching device with a simple equivalent circuit. It can work very fast as it is based on the tunneling of single electrons. Its nanostructure contains a quantum dot island whose material impacts on the device operation. Carbon allotropes such as fullerene (C_60_), carbon nanotubes (CNTs) and graphene nanoscrolls (GNSs) can be utilized as the quantum dot island in SETs. In this study, multiple quantum dot islands such as GNS-CNT and GNS-C_60_ are utilized in SET devices. The currents of two counterpart devices are modeled and analyzed. The impacts of important parameters such as temperature and applied gate voltage on the current of two SETs are investigated using proposed mathematical models. Moreover, the impacts of CNT length, fullerene diameter, GNS length, and GNS spiral length and number of turns on the SET’s current are explored. Additionally, the Coulomb blockade ranges (CB) of the two SETs are compared. The results reveal that the GNS-CNT SET has a lower Coulomb blockade range and a higher current than the GNS-C_60_ SET. Their charge stability diagrams indicate that the GNS-CNT SET has smaller Coulomb diamond areas, zero-current regions, and zero-conductance regions than the GNS-C_60_ SET.

## 1. Introduction

The single electron transistor (SET) is an electronic device that can realize fast switching using nanotechnology [1]. The SET has unique properties such as nanometer size, a simple circuit, and fast operation [2]. The SET contains electrodes and tunnel junctions where a bias voltage is applied between the source and drain electrodes [3]. This single electron device has an island which is located between three electrodes [4]. It can switch an electron from the source electrode to the island and then to the drain electrode through tunnel junctions [5]. This electron transfer cycle is known as single electron tunneling [6]. The electron tunneling is prevented by the Coulomb blockade phenomenon, and then the output current of the SET is equal to zero [7].

In Coulomb blockade conditions, the charging energy (the essential energy for moving an electron to the island) is higher than the thermal energy. Moreover, the thermal resistance is greater than the quantum resistance [8]. In Coulomb blockade conditions, the Fermi energy of the source electrode is lower than the first unoccupied energy level of the quantum dot, so an electron cannot tunnel to the transfer window [9]. The current flow in the SET will be stopped [10]. When the electron overcomes these conditions [11], the electron passes from the tunnel junctions and current can flow in the SET [12].

The SET operation is fast and the speed of electron tunneling depends on the island material, which can be chosen from nanomaterials used in the electronics industry [13]. These materials, such as carbon allotropes and in particular, graphene, have unique properties [14]. These properties affect some of the parameters of the SET and hence its application [15]. Their high carrier mobilities cause fast tunneling of single electrons into the quantum dot island [16]. High electron mobility increases the SET’s speed of operation, which reduces the power consumption and the leakage current in this device [17]. Therefore, utilizing a nanomaterial as the SET island can improve its performance.

**Figure 1:**
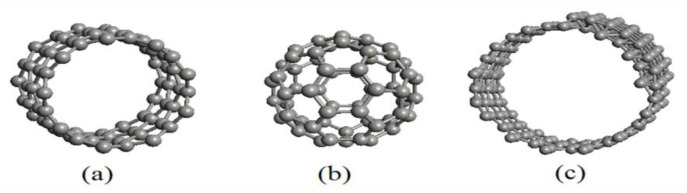
The nanostructures of carbon-based materials: (**a**) carbon nanotube (CNT); (**b**) fullerene (C_60_); (**c**) graphene nanoscroll (GNS).

The SET quantum dot material can be selected from low-dimensional materials such as C_60_ (fullerene) [18], carbon nanotubes (CNTs) [19] and graphene nanoscrolls (GNSs) [20]. Their nanostructures are shown in Figure 1 [21].

The CNT is a one-dimensional material that is cylindrical in shape, as illustrated in Figure 1a [22]. C_60_ is a zero-dimensional material and is spherical in shape, as shown in Figure 1b [23]. The GNS is another form of carbon material which is spiral in shape, as shown in Figure 1c [2,20]. All of the illustrations in Figure 1 were produced with Atomistix ToolKit (ATK) software (Synopsys, Mountain View, CA, USA) [21]. The SET’s island can be designed by utilizing these materials as multiple quantum dots [20,24].

Two different islands were designed using these quantum dots: the first double quantum dot island consisted of a graphene nanoscroll (GNS) and a carbon nanotube (CNT), denoted the GNS-CNT island. The second double quantum dot island included a graphene nanoscroll (GNS) and fullerene (C_60_) and is denoted the GNS-C_60_ island. It is worth noting that the SET can utilize two or more quantum dots as islands [20,24,25].

In this research, the two islands GNS-CNT and GNS-C_60_ were selected and each of them was located in the SET channel. SETs with multiple islands were investigated here for the first time, and hence this research is devoted to their first-principles study. The currents of the GNS-CNT SET and the GNS-C_60_ SET were modeled using mathematical models. The MATLAB codes for the proposed models were implemented to derive their I–V characteristics. The current behavior diagrams were analyzed and compared. Additionally, charge stability diagrams for the two SETs under study were plotted using Atomistix ToolKit (ATK) software (Synopsys, Mountain View, CA, USA) [21], and their Coulomb diamond areas and Coulomb blockade ranges were compared.

It should be noted that SET structures with low-dimensional materials have attracted attention in recent years. For instance, white graphene, as a two-dimensional material, can be utilized in these structures [26]. The SET can be used in various electronic devices such as oscillators [27], sensors [28], detection of gas molecules [29] and single electron memory [30].

## 2. Theoretical Model and Results

### 2.1. The Current Models of SETs

**Figure 2:**
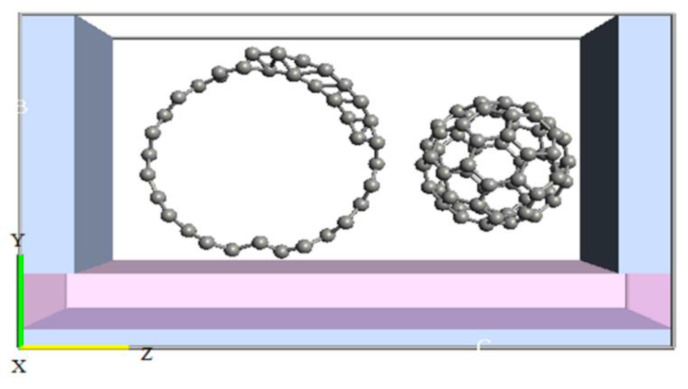
The designed structure for GNS-C_60_ SET.

Single electron transistors (SETs) can switch an electron to achieve the desired current. The electron transfer is based on quantum mechanical effects. The electron passes from different regions of the SET, and consequently the electron wave function changes in each region. The structure of a double quantum dot SET (GNS-CNT SET) was designed as shown in Figure 2 [21].

**Figure 3:**
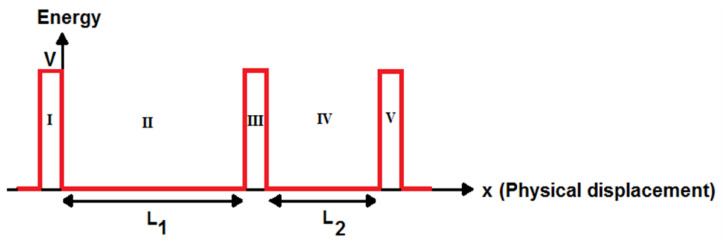
Schematic energy band diagram for GNS-C_60_ SET.

The mathematical model for the current flow is derived for the SET current by solving Schrödinger’s equation and using the Landauer formalism. The SET structure can be divided into different regions. The electron wave function can be found based on Schrödinger’s equation to model its current. The double-island SET can be divided into five parts, consisting of two potential wells with different lengths and three tunnel junctions, for writing Schrödinger’s equations and calculating its transmission coefficient. The schematic energy band diagram for the SET with two different islands is shown in Figure 3.

The SET model is based on two islands such that each island is assumed to behave like a potential well. Moreover, the transmission coefficient of the tunnel barriers is assumed to be one in this current model. Schrödinger’s equation can be derived for the GNS island as follows.

(1) $$\frac{\hbar^{2}}{2m}\frac{\partial^{2}\psi_{I}\left(x\right)}{\partial x^{2}}+\left(E-V\right)\psi_{I}\left(x\right)=0\;x\leq 0\;\mathrm{Region}\;I$$

(2) $$\frac{\hbar^{2}}{2m}\frac{\partial^{2}\psi_{II}\left(x\right)}{\partial x^{2}}+E\psi_{II}\left(x\right)=0\;0<x<L_{1}\;\;\mathrm{Region}\;\mathrm{II}\;$$

(3) $$\frac{\hbar^{2}}{2m}\frac{\partial^{2}\psi_{III}\left(x\right)}{\partial x^{2}}+\left(E-V\right)\psi_{III}\left(x\right)=0\;x\geq L_{1}\;\;\mathrm{Region}\;\mathrm{III}$$

(4) $$\Psi_{I}\left(x\right)=A_{1}e^{k_{1}x}+B_{1\;}e^{-k_{1}x}\;\;\mathrm{where}\;k_{1}=\sqrt{\frac{2m\left(V-E\right)}{\hbar^{2}}}\;\;\;\;$$

(5) $$\Psi_{II}\left(x\right)=A_{2}e^{ik_{2}x}+B_{2\;}e^{-ik_{2}x}\;\;\;\mathrm{where}\;k_{2}=\sqrt{\frac{2mE}{\hbar^{2}}}$$

(6) $$\Psi_{III}\left(x\right)=A_{3}e^{k_{3}x}\;\;\mathrm{where}\;k_{3}=k_{1}=\sqrt{\frac{2m\left(V-E\right)}{\hbar^{2}}}$$

where “ℏ” is the reduced Planck’s constant, “m” is the electron effective mass, $\Psi_{n}\left(x\right),\;n=I,\mathrm{II},\mathrm{III}$ is a wave function, $“L_{1}”$ is the length of the GNS, $k_{n},\;n=1,2,3$ are wave vectors, “E” is the electron energy, “V” is the potential, “x” is the displacement, and $i=\sqrt{-1}$. The coefficients $A_{1},A_{2},A_{3},B_{1,\;}\mathrm{and}\;B_{2}$ are constant. 

Moreover, the boundary conditions from x = 0 to x =$\;\;L_{1}$ for the GNS island are solved as follows:

(7) $$\Psi_{I}\left(0\right)=\Psi_{II}\left(0\right)\;=A_{1}+B_{1\;}=A_{2}+B_{2\;}$$

(8) $$\Psi_{I}{}^{\prime }\left(0\right)=\Psi_{II}{}^{\prime }\left(0\right)=k_{1}A_{1}-k_{1}B_{1\;}=ik_{2}A_{2}-ik_{2}B_{2\;}$$

(9) $$\Psi_{II}\left(L_{1}\right)=\Psi_{III}\left(L_{1}\right)=A_{2}e^{ik_{2}L_{1}}+B_{2\;}e^{-ik_{2}L_{1}}=A_{3}e^{k_{1}L_{1}}$$

(10) $$\Psi_{II}{}^{\prime }\left(L_{1}\right)=\Psi_{III}{}^{\prime }\left(L_{1}\right)=ik_{2}A_{2}e^{ik_{2}L_{1}}-ik_{2\;}B_{2\;}e^{-ik_{2}L_{1}}=k_{1}A_{3}e^{k_{1}L_{1}}$$

where parameters are as defined previously. These equations are utilized for the calculation of the GNS island transmission coefficient. The electron transmission coefficient for the SET with one GNS island is calculated as:

(11) $$T_{GNS}\left(E\right)=\frac{1}{1+K_{GNS\;}{}^{2}sinh^{2}\left(k_{2}L_{1}\right)}$$

(12) $$K_{GNS}=\frac{(\hbar^{2}+ta^{\prime }m)E-\hbar^{2}E_{g_{GNS}}}{2\sqrt{ta^{\prime }\hbar mE\left(E-E_{g_{GNS}}\right)}}$$

where $“L_{1}”$ is the GNS length, $k_{2}=\sqrt{\frac{2mE}{\hbar^{2}}}$, “E” is the electron energy, “m” is the electron effective mass in the GNS, “ℏ” is the reduced Planck’s constant, $a^{\prime }=3a_{c-c_{GNS}}$, $“a_{c-c_{GNS}}”$ is the distance between neighboring carbon atoms in the GNS molecule,$\;“K_{GNS}”$ is the wave vector of GNS,$\;“E_{g_{GNS}}”$ is the GNS bandgap (the energy gap is defined as the difference between the highest occupied and the lowest unoccupied molecular orbitals (HOMO and LUMO)), and “t” is the hopping energy.

Schrödinger’s equation can be derived for the C_60_ island as follows.

(13) $$\frac{\hbar^{2}}{2m}\frac{\partial^{2}\psi_{III}\left(x\right)}{\partial x^{2}}+\left(E-V\right)\psi_{III}\left(x\right)=0\;x\leq 0\;\mathrm{Region}\;\mathrm{III}$$

(14) $$\frac{\hbar^{2}}{2m}\frac{\partial^{2}\psi_{IV}\left(x\right)}{\partial x^{2}}+E\psi_{IV}\left(x\right)=0\;0<x<L_{2}\;\;\mathrm{Region}\;\mathrm{IV}$$

(15) $$\frac{\hbar^{2}}{2m}\frac{\partial^{2}\psi_{V}\left(x\right)}{\partial x^{2}}+\left(E-V\right)\psi_{V}\left(x\right)=0\;x\geq L_{2}\;\;\mathrm{Region}\;V$$

(16) $$\Psi_{III}\left(x\right)=A_{1}e^{k_{1}x}+B_{1\;}e^{-k_{1}x}\;\mathrm{where}\;k_{1}=\sqrt{\frac{2m\left(V-E\right)}{\hbar^{2}}}$$

(17) $$\Psi_{IV}\left(x\right)=A_{2}e^{ik_{2}x}+B_{2\;}e^{-ik_{2}x}\;\mathrm{where}\;k_{2}=\sqrt{\frac{2mE}{\hbar^{2}}}$$

(18) $$\Psi_{V}\left(x\right)=A_{3}e^{k_{3}x}\;\;\mathrm{where}\;k_{3}=k_{1}=\sqrt{\frac{2m\left(V-E\right)}{\hbar^{2}}}\;$$

where $“L_{2}”$ is the fullerene (C_60_) diameter, and $\Psi_{n}\left(x\right),\;n=\mathrm{III},\;\mathrm{IV},V$ is the electron wave function. Other parameters are as defined previously.

The boundary conditions from x = 0 to x =$\;L_{2}$ for the C_60_ island are solved as follows:

(19) $$\Psi_{III}\left(0\right)=\Psi_{IV}\left(0\right)\;=A_{1}+B_{1\;}=A_{2}+B_{2\;}$$

(20) $$\Psi_{III}{}^{\prime }\left(0\right)=\Psi_{IV}{}^{\prime }\left(0\right)=k_{1}A_{1}-k_{1}B_{1\;}=ik_{2}A_{2}-ik_{2}B_{2\;}$$

(21) $$\Psi_{IV}\left(L_{2}\right)=\Psi_{V}\left(L_{2}\right)=A_{2}e^{ik_{2}L_{2}}+B_{2\;}e^{-ik_{2}L_{2}}=A_{3}e^{k_{1}L_{2}}$$

(22) $$\Psi_{IV}{}^{\prime }\left(L_{2}\right)=\Psi_{V}{}^{\prime }\left(L_{2}\right)=ik_{2}A_{2}e^{ik_{2}L_{2}}-k_{2\;}B_{2\;}e^{-k_{2}L_{2}}=k_{1}A_{3}e^{k_{1}L_{2}}$$

These equations are solved and the transmission coefficient for the fullerene (C_60_) island is calculated as follows:

(23) $$T_{C60}\left(E\right)=\frac{1}{1+K_{C60}{}^{2}sinh^{2}\left(k_{2}L_{2}\right)}$$

(24) $$K_{C60}=\frac{(h^{2}+ta^{\prime \prime }m)E-\hbar^{2}E_{g_{C60}}}{2\sqrt{ta^{\prime \prime }\hbar mE\left(E-E_{g_{C60}}\right)}}$$

where $“L_{2}”$ is the fullerene (C_60_) diameter,$\;a^{\prime \prime }=3a_{c-c_{C60}}$, $“a_{c-c_{C60}}”$ is the distance between neighboring carbon atoms in the C_60_ molecule,$“K_{C60}”$ is the wave vector of C_60_, and $“E_{g_{C60}}”$ is the C_60_ bandgap. Other parameters are as defined previously.

The transmission coefficient of the tunnel barriers is assumed to be one. The transmission coefficient of a GNS-C_60_ SET is:

(25) $$T_{1}\left(E\right)=\left(T_{GNS}\left(E\right)\cdot T_{C60}\left(E\right)\right)$$

where $“T_{GNS}\left(E\right)”$ is the transmission coefficient of the GNS island and $“T_{C60}\left(E\right)”$ is the transmission coefficient of the C_60_ island. The SET current based on the Landauer formalism depends on the transmission coefficient “T(E)” and the Fermi probability function “F(E)”, which is given by:

(26) $$I=\overset{\eta }{\underset{0}{\int }}T_{1}\left(E\right)\cdot F\left(E\right)dE\;$$

where $“T_{1}\left(E\right)”$ is the transmission coefficient, $\eta =\frac{E_{F}-E_{g}}{K_{B}T},$ $“E_{F}”$ is the Fermi energy,$“E_{g}”$ is the band gap energy, $“k_{B}”$ is Boltzmann’s constant, and “T” is the temperature. “F(E)” is the Fermi probability function which is defined as:

(27) $$F\left(E\right)=\frac{1}{\exp\left(\frac{E-E_{F}}{k_{B}T}\right)+1}$$

where “E” is the electron energy. Then, the transmission coefficient of a GNS-C_60_ SET is calculated as:

(28) $$\begin{array}{l} T_{1}\left(E\right)\\ =(1+{\left(\frac{\frac{4E}{3ta}\left(\frac{t^{2}n_{1}^{2}a+3aL_{1}{}^{2}}{3tn_{1}^{2}a^{2}}\right)-2{\left(\frac{t^{2}n_{1}^{2}a+3aL_{1}{}^{2}}{3tn_{1}^{2}a^{2}}\right)}^{2}+\frac{4E}{3ta}\left(\frac{t^{2}n_{2}^{2}a+3aL_{1}{}^{2}}{3tn_{2}^{2}a^{2}}\right)-2{\left(\frac{t^{2}n_{2}^{2}a+3aL_{1}{}^{2}}{3tn_{2}^{2}a^{2}}\right)}^{2}}{2\sqrt{\frac{4E}{3ta}\left(\frac{t^{2}n_{1}^{2}a+3aL_{1}{}^{2}}{3tn_{1}^{2}a^{2}}\right)-2{\left(\frac{t^{2}n_{1}^{2}a+3aL_{1}{}^{2}}{3tn_{1}^{2}a^{2}}\right)}^{2}}\sqrt{\frac{4E}{3ta}\left(\frac{t^{2}n_{2}^{2}a+3aL_{1}{}^{2}}{3tn_{2}^{2}a^{2}}\right)-2{\left(\frac{t^{2}n_{2}^{2}a+3aL_{1}{}^{2}}{3tn_{2}^{2}a^{2}}\right)}^{2}}}\right)}^{2}\\ \times sinh^{2}\left(L^{\prime }\sqrt{\frac{4E}{3ta}\left(\frac{t^{2}n_{2}^{2}a+3aL_{1}{}^{2}}{3tn_{2}^{2}a^{2}}\right)-2{\left(\frac{t^{2}n_{2}^{2}a+3aL_{1}{}^{2}}{3tn_{2}^{2}a^{2}}\right)}^{2}}\right){)}^{-1}\\ \times \frac{AK_{B}Tx\left(K_{B}Tx+Eg_{C60}\right)}{AK_{B}Tx\left(K_{B}Tx+Eg_{C60}\right)+{\left(B\left(K_{B}Tx+Eg_{C60}\right)+CAK_{B}Tx\right)}^{2}{\left[{\left(B\left(K_{B}Tx+Eg_{C60}\right)L_{2}{}^{2}\right)}^{\frac{1}{2}}+\frac{{\left(B\left(K_{B}Tx+Eg_{C60}\right)L_{2}{}^{2}\right)}^{\frac{3}{2}}}{6}\right]}^{2}} \end{array}$$

where “E” is the electron energy, “t” is the nearest-neighbor C–C tight-binding overlap energy,$\;“n_{1}”$ and $“n_{2}”$ are values of the chirality number, “a” is the starting value of θ, and θ is the rolling angle of the GNS. Furthermore $“L^{\prime }\;”$ is the GNS spiral length and $“L_{1}”$ represents the GNS length. Moreover, $“E_{gc60}”$ is the energy band gap of fullerene,$“K_{B}”$ is Boltzmann’s constant, and “T” is the temperature. Furthermore,$\;x=\frac{E-E_{g}}{K_{B}T},$ $A=\frac{16m}{8.1\hbar a_{c-c}}$, $B=\left(\frac{2m}{\hbar }\right)$, and $C=\left(\frac{2}{8.1a_{c-c}}\right).$ In addition, “m” is equal to the electron effective mass, “ ℏ” is the reduced Planck’s constant, $a_{c-c}=1.42A{}^{\circ}$ is the carbon–carbon bond length, and $“L_{2}”$ is the C_60_ diameter.

The transmission coefficient for a GNS-C_60_ SET and the Landauer formalism are utilized for the drain–source current modeling of this nanoscale device as follows:

(29) $$\begin{array}{l} I_{ds_{1}}\\ =\overset{\eta }{\underset{0}{\int }}(1+{\left(\frac{\frac{4E}{3ta}\left(\frac{t^{2}n_{1}^{2}a+3aL_{1}{}^{2}}{3tn_{1}^{2}a^{2}}\right)-2{\left(\frac{t^{2}n_{1}^{2}a+3aL_{1}{}^{2}}{3tn_{1}^{2}a^{2}}\right)}^{2}+\frac{4E}{3ta}\left(\frac{t^{2}n_{2}^{2}a+3aL_{1}{}^{2}}{3tn_{2}^{2}a^{2}}\right)-2{\left(\frac{t^{2}n_{2}^{2}a+3aL_{1}{}^{2}}{3tn_{2}^{2}a^{2}}\right)}^{2}}{2\sqrt{\frac{4E}{3ta}\left(\frac{t^{2}n_{1}^{2}a+3aL_{1}{}^{2}}{3tn_{1}^{2}a^{2}}\right)-2{\left(\frac{t^{2}n_{1}^{2}a+3aL_{1}{}^{2}}{3tn_{1}^{2}a^{2}}\right)}^{2}}\sqrt{\frac{4E}{3ta}\left(\frac{t^{2}n_{2}^{2}a+3aL_{1}{}^{2}}{3tn_{2}^{2}a^{2}}\right)-2{\left(\frac{t^{2}n_{2}^{2}a+3aL_{1}{}^{2}}{3tn_{2}^{2}a^{2}}\right)}^{2}}}\right)}^{2}\\ \times sinh^{2}\left(L^{\prime }\sqrt{\frac{4E}{3ta}\left(\frac{t^{2}n_{2}^{2}a+3aL_{1}{}^{2}}{3tn_{2}^{2}a^{2}}\right)-2{\left(\frac{t^{2}n_{2}^{2}a+3aL_{1}{}^{2}}{3tn_{2}^{2}a^{2}}\right)}^{2}}\right){)}^{-1}\\ \times \frac{AK_{B}Tx\left(K_{B}Tx+Eg_{C60}\right)}{AK_{B}Tx\left(K_{B}Tx+Eg_{C60}\right)+{\left(B\left(K_{B}Tx+Eg_{C60}\right)+CAK_{B}Tx\right)}^{2}{\left[{\left(B\left(K_{B}Tx+Eg_{C60}\right)L_{2}{}^{2}\right)}^{\frac{1}{2}}+\frac{{\left(B\left(K_{B}Tx+Eg_{C60}\right)L_{2}{}^{2}\right)}^{\frac{3}{2}}}{6}\right]}^{2}}\cdot \frac{dE}{e^{x-\eta }+1} \end{array}$$

where $\eta =\frac{E_{F}-E_{gC60}}{K_{B}T}$ and $“E_{F}”$ is the Fermi level of the islands. The other parameters are as defined previously.

**Figure 4:**
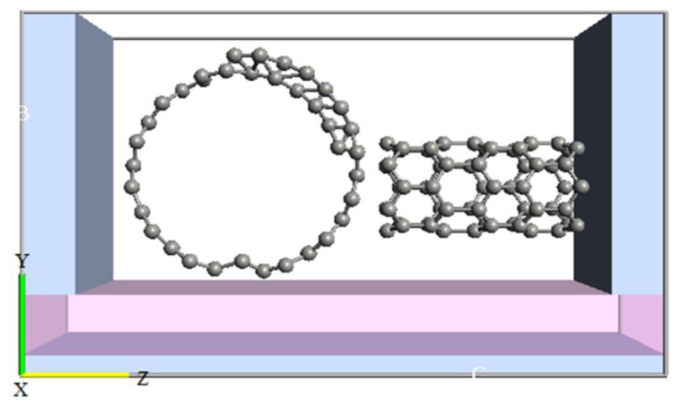
The designed structure for GNS-CNT SET.

The second structure in our study was a GNS-CNT SET, which was designed with ATK software and is shown in Figure 4 [21].

**Figure 5:**
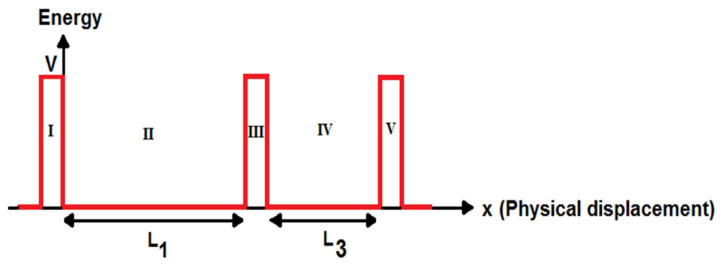
Schematic for energy band diagram of GNS-CNT SET.

The total transmission of this GNS-CNT SET and its current using the aforementioned calculations are modeled as follows. The SET is divided into five parts, and these regions are shown in Figure 5.

Schrödinger’s equation is written for the CNT island as:

(30) $$\frac{\hbar^{2}}{2m}\frac{\partial^{2}\psi_{III}\left(x\right)}{\partial x^{2}}+\left(E-V\right)\psi_{III}\left(x\right)=0\;x\leq 0\;\mathrm{Region}\;\mathrm{III}$$

(31) $$\frac{\hbar^{2}}{2m}\frac{\partial^{2}\psi_{IV}\left(x\right)}{\partial x^{2}}+E\psi_{IV}\left(x\right)=0\;0<x<L_{3\;\;}\;\mathrm{Region}\;\mathrm{IV}$$

(32) $$\frac{\hbar^{2}}{2m}\frac{\partial^{2}\psi_{V}\left(x\right)}{\partial x^{2}}+\left(E-V\right)\psi_{V}\left(x\right)=0\;x\geq L_{3}\;\mathrm{Region}\;V$$

(33) $$\Psi_{III}\left(x\right)=A_{1}e^{k_{1}x}+B_{1\;}e^{-k_{1}x}\;\;\mathrm{where}\;k_{1}=\sqrt{\frac{2m\left(V-E\right)}{\hbar^{2}}}$$

(34) $$\Psi_{IV}\left(x\right)=A_{2}e^{ik_{2}x}+B_{2\;}e^{-ik_{2}x}\;\;\mathrm{where}\;k_{2}=\sqrt{\frac{2mE}{\hbar^{2}}}$$

(35) $$\Psi_{V}\left(x\right)=A_{3}e^{k_{3}x}\;\;\mathrm{where}\;k_{3}=k_{1}=\sqrt{\frac{2m\left(V-E\right)}{\hbar^{2}}}$$

where $“L_{3}”$ is the length of the CNT and the other parameters are as defined previously. The boundary conditions from x = 0 to x= $L_{3}$ for an island are solved as follows:

(36) $$\Psi_{III}\left(0\right)=\Psi_{IV}\left(0\right)\;=A_{1}+B_{1\;}=A_{2}+B_{2\;}$$

(37) $$\Psi_{III}{}^{\prime }\left(0\right)=\Psi_{IV}{}^{\prime }\left(0\right)=k_{1}A_{1}-k_{1}B_{1\;}=ik_{2}A_{2}-ik_{2}B_{2\;}$$

(38) $$\Psi_{IV}\left(L_{3}\right)=\Psi_{V}\left(L_{3}\right)=A_{2}e^{ik_{2}L_{3}}+B_{2\;}e^{-ik_{2}L_{3}}=A_{3}e^{k_{1}L_{3}}$$

(39) $$\Psi_{IV}{}^{\prime }\left(L_{3}\right)=\Psi_{IV}{}^{\prime }\left(L_{3}\right)=ik_{2}A_{2}e^{ik_{2}L_{3}}-ik_{2\;}B_{2\;}e^{-ik_{2}L_{3}}=k_{1}A_{3}e^{k_{1}L_{3}}$$

The transmission coefficient of the SET with a single carbon nanotube (CNT) island is calculated as:

(40) $$T_{CNT}\left(E\right)=\frac{1}{1+K_{CNT}{}^{2}sinh^{2}\left(k_{2}L_{3}\right)}$$

(41) $$K_{CNT}=\frac{(\hbar^{2}+ta^{\prime \prime \prime }m)E-\hbar^{2}E_{g_{CNT}}}{2\sqrt{ta^{\prime \prime \prime }\hbar mE\left(E-E_{g_{CNT}}\right)}}$$

where $“L_{3}”$ is the CNT length, $a^{\prime \prime \prime }=3a_{c-c_{CNT}}$, $“a_{c-c_{CNT}}”$ is the distance between neighboring carbon atoms in the CNT molecule,$\;“K_{CNT}”$ is the wave vector of the CNT, $“E_{g_{CNT}}”$ is the CNT bandgap, and “t” is the hopping energy. The transmission coefficient of the GNS-CNT SET is:

(42) $$T_{2}\left(E\right)=\left(T{\left(E\right)}_{GNS}\cdot T{\left(E\right)}_{CNT}\right)$$

where $“T_{GNS}\left(E\right)”$ is the transmission coefficient of the GNS island and $“T_{CNT}\left(E\right)”$ is the transmission coefficient of the CNT island.

The transmission coefficient and current of the GNS-CNT SET can be modeled as follows:

(43) $$\begin{array}{l} T_{2}\left(E\right)\\ =(1+{\left(\frac{\frac{4E}{3ta}\left(\frac{t^{2}n_{1}^{2}a+3aL_{1}{}^{2}}{3tn_{1}^{2}a^{2}}\right)-2{\left(\frac{t^{2}n_{1}^{2}a+3aL_{1}{}^{2}}{3tn_{1}^{2}a^{2}}\right)}^{2}+\frac{4E}{3ta}\left(\frac{t^{2}n_{2}^{2}a+3aL_{1}{}^{2}}{3tn_{2}^{2}a^{2}}\right)-2{\left(\frac{t^{2}n_{2}^{2}a+3aL_{1}{}^{2}}{3tn_{2}^{2}a^{2}}\right)}^{2}}{2\sqrt{\frac{4E}{3ta}\left(\frac{t^{2}n_{1}^{2}a+3aL_{1}{}^{2}}{3tn_{1}^{2}a^{2}}\right)-2{\left(\frac{t^{2}n_{1}^{2}a+3aL_{1}{}^{2}}{3tn_{1}^{2}a^{2}}\right)}^{2}}\sqrt{\frac{4E}{3ta}\left(\frac{t^{2}n_{2}^{2}a+3aL_{1}{}^{2}}{3tn_{2}^{2}a^{2}}\right)-2{\left(\frac{t^{2}n_{2}^{2}a+3aL_{1}{}^{2}}{3tn_{2}^{2}a^{2}}\right)}^{2}}}\right)}^{2}\\ \times sinh^{2}\left(L^{\prime }\sqrt{\frac{4E}{3ta}\left(\frac{t^{2}n_{2}^{2}a+3aL_{1}{}^{2}}{3tn_{2}^{2}a^{2}}\right)-2{\left(\frac{t^{2}n_{2}^{2}a+3aL_{1}{}^{2}}{3tn_{2}^{2}a^{2}}\right)}^{2}}\right){)}^{-1}\\ \times \frac{AK_{B}Tx\left(K_{B}Tx+Eg_{CNT}\right)}{AK_{B}Tx\left(K_{B}Tx+Eg_{CNT}\right)+{\left(B\left(K_{B}Tx+Eg_{CNT}\right)+CAK_{B}Tx\right)}^{2}{\left[{\left(B\left(K_{B}Tx+Eg_{CNT}\right)L_{3}{}^{2}\right)}^{\frac{1}{2}}+\frac{{\left(B\left(K_{B}Tx+Eg_{CNT}\right)L_{3}{}^{2}\right)}^{\frac{3}{2}}}{6}\right]}^{2}} \end{array}$$

where $“E_{gCNT}”$ is the energy bandgap of the CNT and “$\;L_{3}$” is the CNT length. Other parameters are as defined previously. The Landauer formalism can again be utilized to model the drain–source current of this double quantum dot SET device:

(44) $$\begin{array}{l} I_{ds_{2}}\\ =\overset{\mu }{\underset{0}{\int }}(1+{\left(\frac{\frac{4E}{3ta}\left(\frac{t^{2}n_{1}^{2}a+3aL_{1}{}^{2}}{3tn_{1}^{2}a^{2}}\right)-2{\left(\frac{t^{2}n_{1}^{2}a+3aL_{1}{}^{2}}{3tn_{1}^{2}a^{2}}\right)}^{2}+\frac{4E}{3ta}\left(\frac{t^{2}n_{2}^{2}a+3aL_{1}{}^{2}}{3tn_{2}^{2}a^{2}}\right)-2{\left(\frac{t^{2}n_{2}^{2}a+3aL_{1}{}^{2}}{3tn_{2}^{2}a^{2}}\right)}^{2}}{2\sqrt{\frac{4E}{3ta}\left(\frac{t^{2}n_{1}^{2}a+3aL_{1}{}^{2}}{3tn_{1}^{2}a^{2}}\right)-2{\left(\frac{t^{2}n_{1}^{2}a+3aL_{1}{}^{2}}{3tn_{1}^{2}a^{2}}\right)}^{2}}\sqrt{\frac{4E}{3ta}\left(\frac{t^{2}n_{2}^{2}a+3aL_{1}{}^{2}}{3tn_{2}^{2}a^{2}}\right)-2{\left(\frac{t^{2}n_{2}^{2}a+3aL_{1}{}^{2}}{3tn_{2}^{2}a^{2}}\right)}^{2}}}\right)}^{2}\\ \times sinh^{2}\left(L^{\prime }\sqrt{\frac{4E}{3ta}\left(\frac{t^{2}n_{2}^{2}a+3aL_{1}{}^{2}}{3tn_{2}^{2}a^{2}}\right)-2{\left(\frac{t^{2}n_{2}^{2}a+3aL_{1}{}^{2}}{3tn_{2}^{2}a^{2}}\right)}^{2}}\right){)}^{-1}\\ \times \frac{AK_{B}Tx\left(K_{B}Tx+Eg_{CNT}\right)}{AK_{B}Tx\left(K_{B}Tx+Eg_{CNT}\right)+{\left(B\left(K_{B}Tx+Eg_{CNT}\right)+CAK_{B}Tx\right)}^{2}{\left[{\left(B\left(K_{B}Tx+Eg_{CNT}\right)L_{3}{}^{2}\right)}^{\frac{1}{2}}+\frac{{\left(B\left(K_{B}Tx+Eg_{CNT}\right)L_{3}{}^{2}\right)}^{\frac{3}{2}}}{6}\right]}^{2}}\cdot \frac{dE}{e^{x-\eta }+1\;} \end{array}$$

where $x=\frac{E-E_{g}}{K_{B}T}$,$\;E_{g}=\frac{E_{gGNS}+E_{gCNT}}{2}$, “E” is the electron energy level, $\eta =\frac{E_{F}-E_{gCNT}}{K_{B}T}$, and $“E_{F}”$ is the Fermi level of the islands.

### 2.2. Results and Discussion

**Figure 6:**
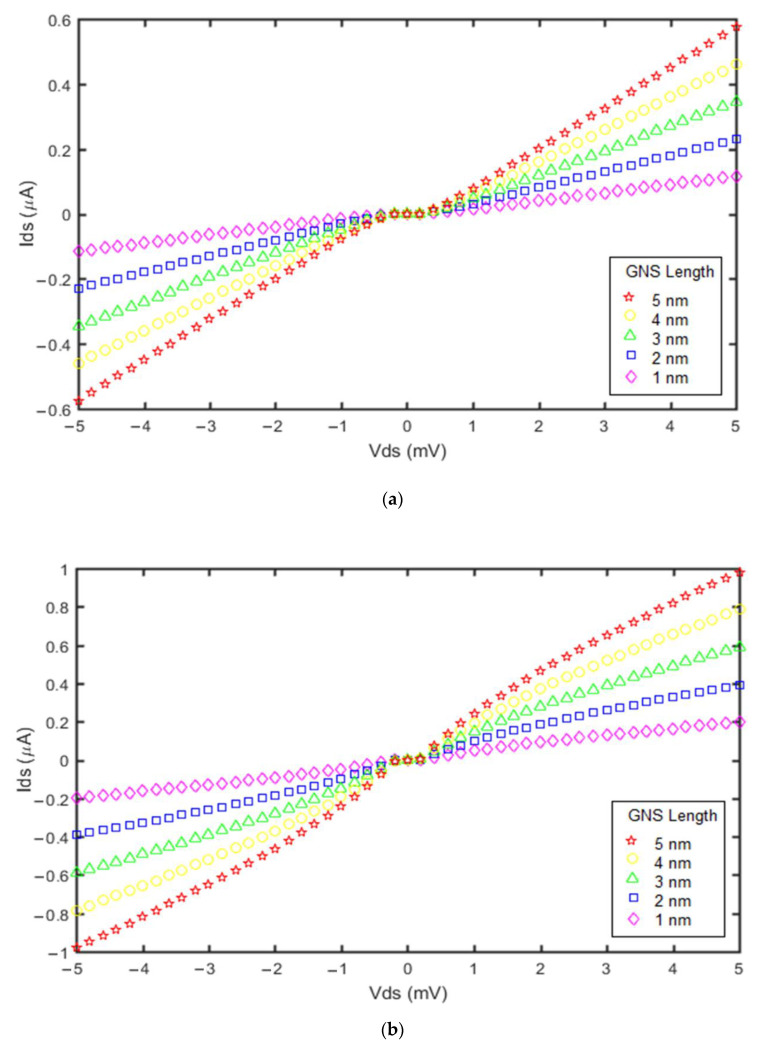
Current vs. voltage diagrams obtained from the proposed models for different GNS lengths: (**a**) GNS-C_60_ SET; (**b**) GNS-CNT SET. For both devices, the applied gate voltage was 1 mV and the temperature was 300 °K.

Based on the proposed models, the effect of the GNS length on the current of the GNS-C_60_ SET and the GNS-CNT SET was investigated, as shown in Figure 6.

Analysis of the curves in Figure 6a,b indicates that when the GNS length increased from 1 nm to 5 nm, the current of both devices (GNS-C_60_ SET and GNS-CNT SET) increased. The highest GNS length has the highest output current and also has the lowest Coulomb blockade range and zero-current range in the two diagrams in Figure 6. Both transistors had the highest current for a GNS length of 5 nm. This is due to the fact that in the proposed current models, thinner tunnel barriers exist with larger-sized GNS islands. The thinner tunnel barriers cause a reduction in the co-tunneling electrons to the island and reduce the Coulomb blockade range, as can be seen in Figure 6a,b. Moreover, the comparative study of these figures reveals the fact that the impact of GNS length variation on the device current was more significant in the GNS-CNT SET than the GNS-C_60_ SET.

**Figure 7:**
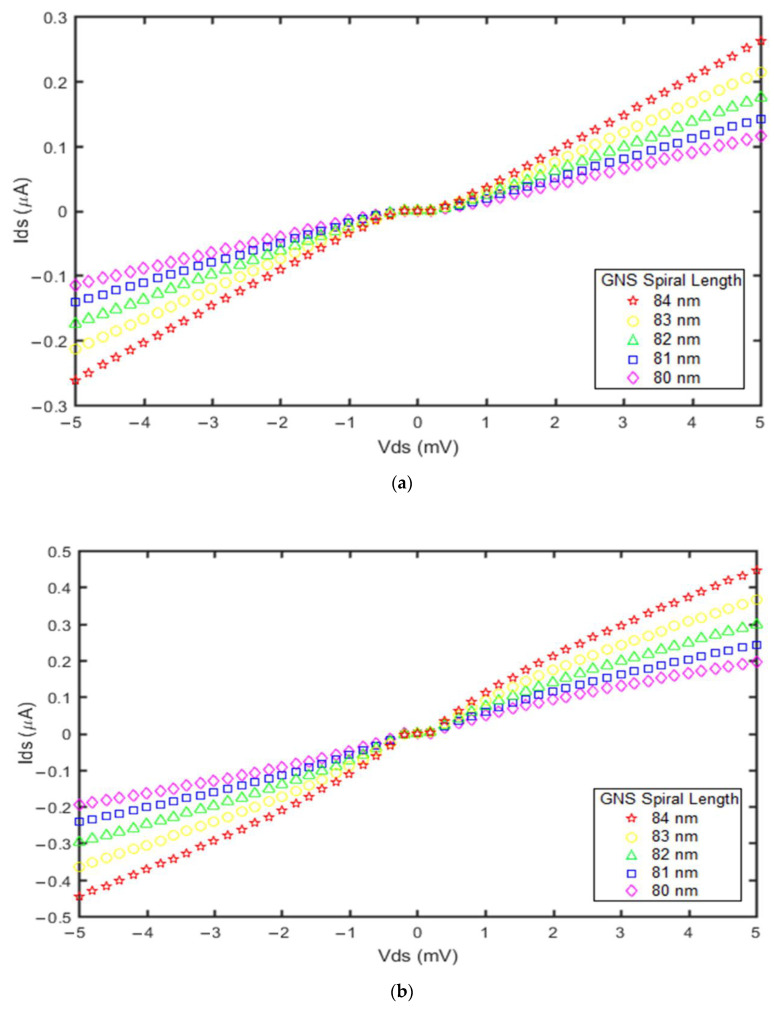
Current vs. voltage diagrams obtained from the proposed models for different GNS spiral lengths: (**a**) GNS-C_60_ SET; (**b**) GNS-CNT SET. For both devices, the applied gate voltage was 1 mV and the temperature was 300 °K.

The impact of GNS spiral length was also investigated in the GNS-C_60_ SET and GNS-CNT SET devices. Based on the proposed current models, the current versus voltage diagrams were extracted and plotted, as shown in Figure 7.

By increasing the GNS spiral length from 80 nm to 84 nm with a constant number of turns, the tunnel barrier thickness was decreased. Electron tunneling through thinner tunnel barriers results in higher speed of electron transfer. Therefore, the Coulomb blockade and zero-current ranges decreased in both devices. From Figure 7a,b it is seen that the SETs have the highest current for a GNS spiral length of 84 nm. Comparison of these diagrams indicates that the effect of the GNS spiral length on the current ranges in the GNS-CNT SET is greater than in the GNS-C_60_ SET. Furthermore, its Coulomb blockade range is less than for the GNS-C_60_ SET. Thus, the GNS-CNT SET current is higher than that of the GNS-C_60_ SET.

**Figure 8:**
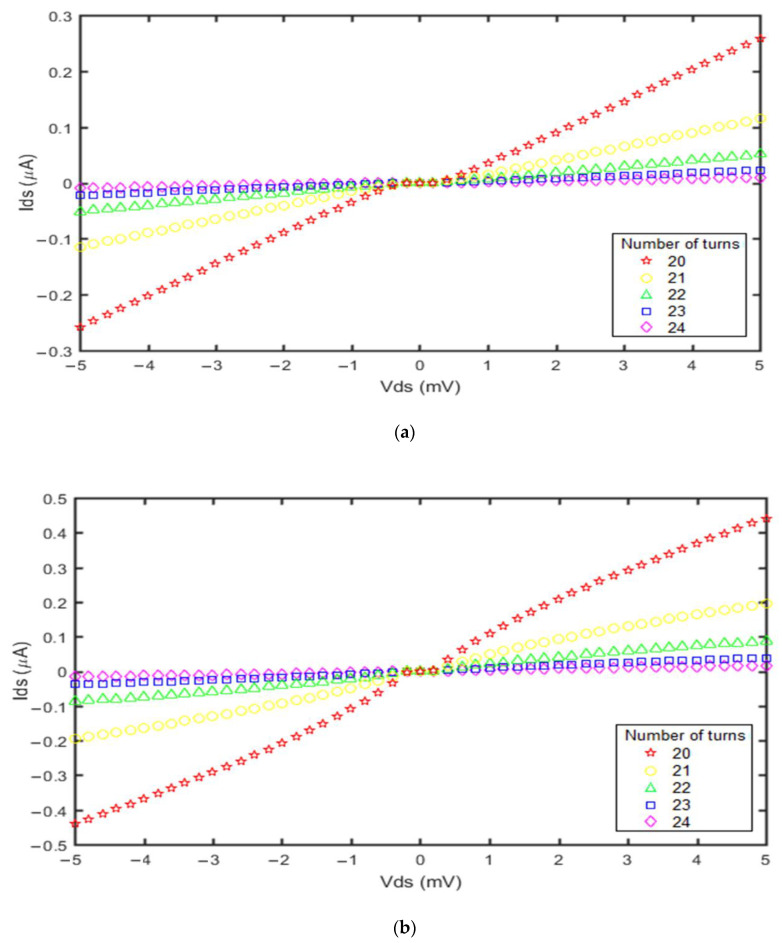
Current vs. voltage diagrams obtained from the proposed models for different GNS numbers of turns: (**a**) GNS-C_60_ SET; (**b**) GNS-CNT SET. For both devices, the applied gate voltage was 1 mV and the temperature was 300 °K.

The number of turns in the GNS influences the SET current, as shown in Figure 8.

The number of turns in the GNS has a direct impact on the SET operation, as seen in Figure 8. We changed the number of turns in the GNS of both devices from 20 to 24 turns. It was revealed that the impact of the number of turns on the current in the GNS-CNT device was greater than for the GNS-C_60_ device. The analysis also indicated that the lowest number of turns in the GNS resulted in increased currents in the two nanoscale transistors. This also decreased their Coulomb blockade ranges towards the smallest values. The lowest number of turns corresponded with the largest island size, which had the thinnest tunnel barriers. Furthermore, the number of available states increases, and the single electron tunnels faster to the island, leading to the device operating with higher speed. On the other hand, when the number of turns in the GNS increased while the GNS spiral length remained constant, the GNS length became smaller. Therefore, the number of available states for electron tunneling increases, causing the electron tunneling speed to increase. These results are in agreement with the investigation of GNS length variation shown in Figure 6.

**Figure 9:**
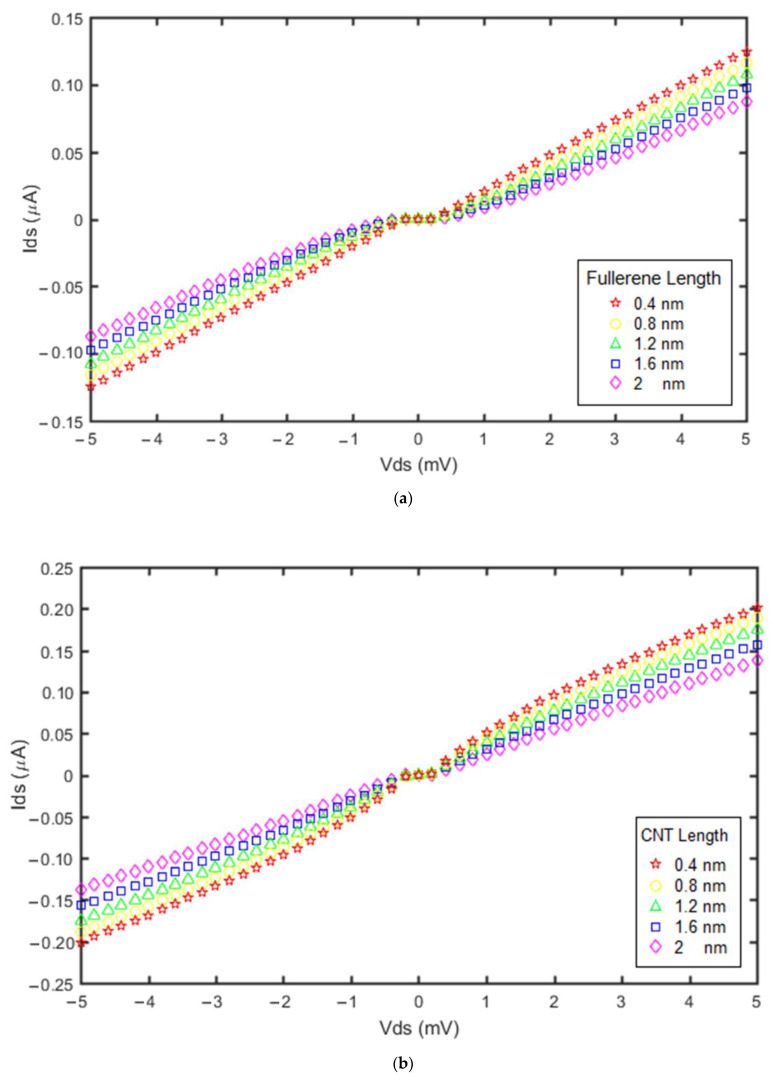
Current vs. voltage diagrams obtained from the proposed models for: (**a**) different fullerene lengths in GNS-C_60_ SET; (**b**) different CNT lengths in GNS-CNT SET. For both devices, the applied gate voltage was 1 mV and the temperature was 300 °K.

Based on our proposed models, the currents for the GNS-C_60_ SET and the GNS-CNT SET were investigated and plotted in Figure 9 for different CNT lengths and fullerene lengths.

The lengths of the two quantum dots varied between 0.4 nm and 2 nm. The results show that decreasing the fullerene length and CNT length increased the current of both devices. Moreover, these electronic devices with lower quantum dot lengths have a higher current but the Coulomb blockade range presents no significant change. However, the Coulomb blockade range in the GNS-CNT SET was less than in the GNS-fullerene SET.

**Figure 10:**
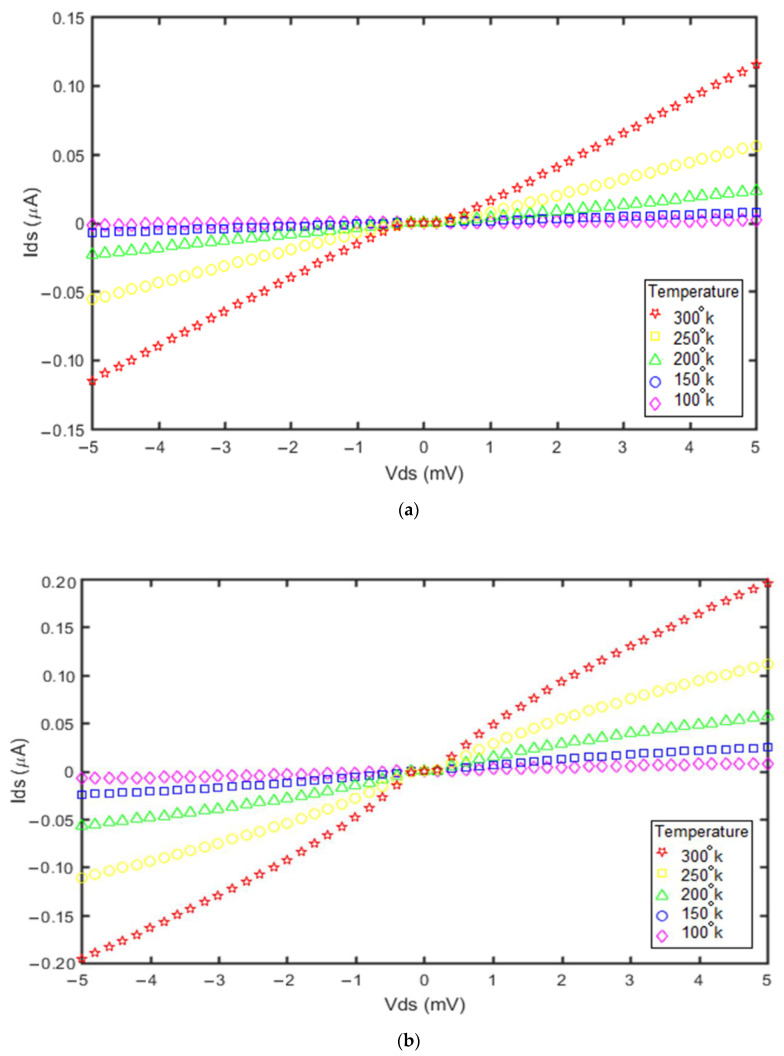
Impact of temperature on the device current: (**a**) GNS-C_60_ SET; (**b**) GNS-CNT SET.

Temperature is another factor affecting the SET current that could be investigated using the proposed model. The drain–source current versus drain–source voltage plots associated with different temperatures are shown in Figure 10.

The ambient temperature has a direct effect on the current of both devices. The curves in Figure 10a,b indicate that increasing the ambient temperature from 100 °K to 300 °K increased the SET current significantly. The maximum current was at 300 °K in the two SETs, but the current of the GNS-CNT device was greater than that of the GNS-C_60_ device. Therefore, the impact of ambient temperature on the SET current with the GNS-CNT island was greater than for the GNS-C_60_ SET. In addition, increasing the temperature decreased the Coulomb blockade ranges, as confirmed in Figure 10a,b.

**Figure 11:**
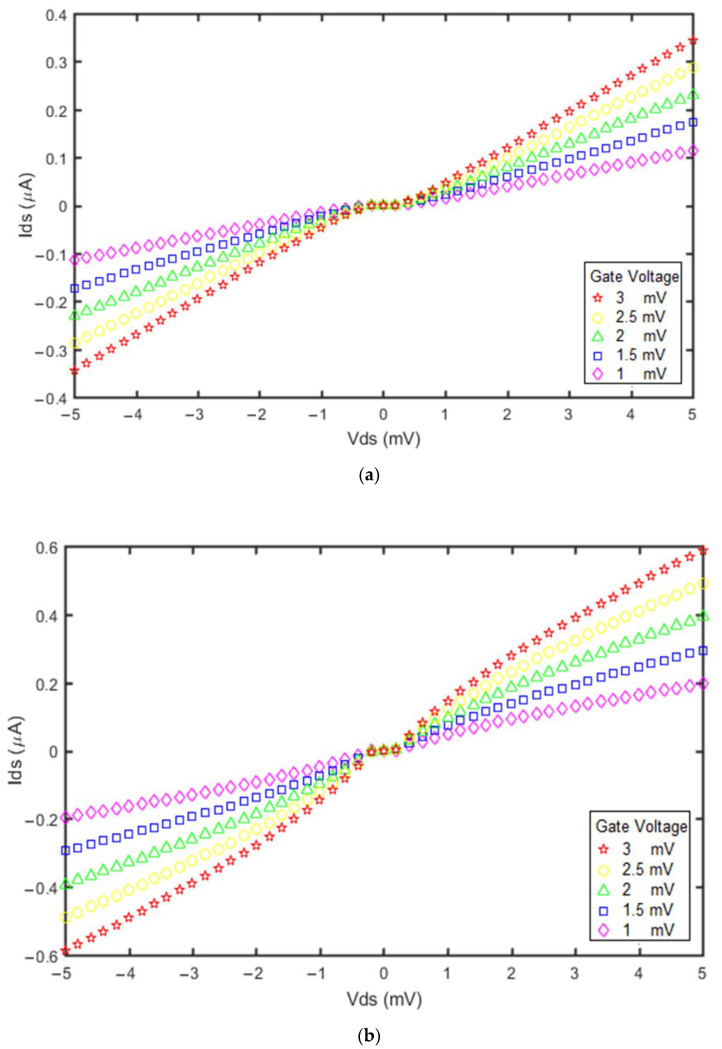
Impact of gate voltage on the device current at T = 300 °K: (**a**) GNS-C_60_ SET; (**b**) GNS-CNT SET.

Another factor affecting SET operation is the applied gate voltage, as shown in Figure 11. 

Figure 11 shows the current versus voltage plots for both devices when the gate voltage varies from 1 mV to 3 mV. The currents of both devices increased with increasing applied gate voltage. The increase in the gate voltage shifts the first unoccupied energy level to a lower level. Therefore, the single electron needs to lower its energy to move towards the transfer window and can tunnel to the island with higher speed. Moreover, comparison of Figure 11a,b indicates that changing the gate voltage has only a small impact on the Coulomb blockade range. However, its effect on the Coulomb blockade range and current of the GNS-CNT SET was greater than for the GNS-C_60_ SET.

**Figure 12:**
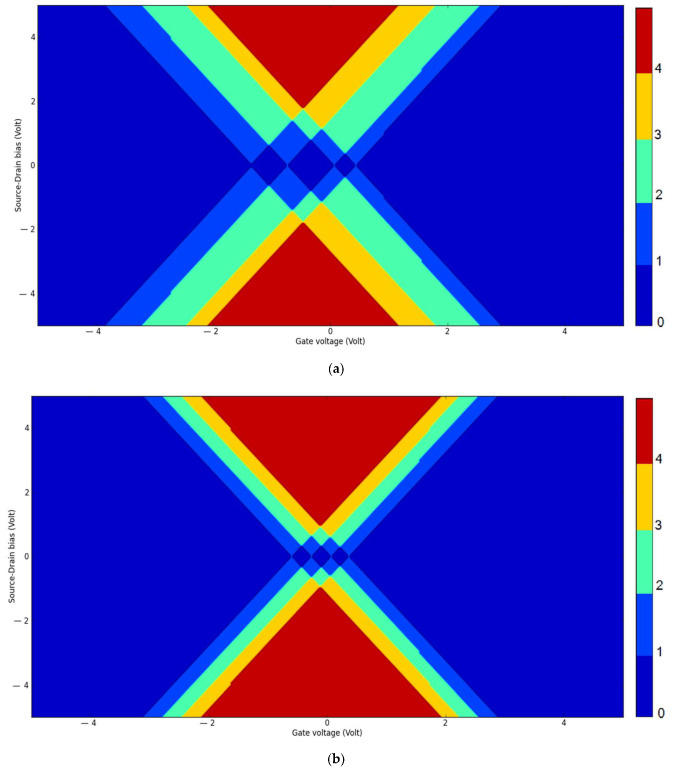
The charge stability diagrams for double quantum dot islands with 96 carbon atoms: (**a**) GNS- C_60_ island; (**b**) GNS- CNT island. (The color bar on the right side represents the corresponding charge states in the diagram).

The island material affects the Coulomb blockade and zero-conductance region of SETs. The Coulomb blockade is a diamond-like region in the charge stability diagram. The charge stability diagram is a function of the source–drain voltage and the gate voltage which can be extracted and plotted with the aid of ATK software [21]. The two islands with equal numbers of atoms (GNS-C_60_ and GNS-CNT) were designed with ATK software using xyz coordinates. The DFT method using a local-density approximation (LDA) was selected for the simulation. The resulting charge stability diagrams are plotted in Figure 12. The corresponding color bar beside each part represents different charge states in these diagrams.

To evaluate the Coulomb blockade regions and to calculate Coulomb diamond areas in these charge stability diagrams in a quantitative manner, key parameters were extracted from Figure 12, and these data are reported in Table 1.

The stability diagrams in Figure 12a,b show that the SET with the GNS-CNT island had smaller Coulomb diamonds than the SET with the GNS-C_60_ island. Comparison of the data given in Table 1 indicates that the total Coulomb diamond area for the GNS-CNT device was smaller than for the GNS-C_60_ device. Moreover, the zero-voltage regions in the GNS-CNT island were smaller than in the GNS-C_60_ island. Therefore, the conductance of the GNS-CNT island was greater than that of the GNS-C_60_ island. The higher conductance could be due to the shape of the GNS-CNT island.

## 3. Conclusions

Single electron transistors (SETs) are fast electronic devices that can be utilized in many electronic devices such as oscillators, sensors, detection of gas molecules, and single electron memory. Recently, nanomaterials have been used in electronic device applications. Therefore, these materials were utilized in SETs in this study with the aim of device performance improvement. The material of the SET island can play a significant role in SET operation and in mitigating the limitations on its operation associated with the leakage current and power consumption. Fullerene (C_60_) or a carbon nanotube (CNT) were utilized with a graphene nanoscroll (GNS) in our study. Double quantum dot island devices such as GNS-C_60_ and GNS-CNT were investigated, and the SET current was explored using two mathematical models for each device. The current models were derived based on solving Schrödinger’s equation and using the Landauer formalism. The current versus drain–source voltage diagrams for the two SETs were extracted and plotted for comparison. The impacts of GNS length, number of turns and spiral length were explored in both devices. Moreover, the effect of temperature and gate voltage on the operation of the devices was studied. The results revealed the fact that the Coulomb blockade range (CB) and zero-current range in the GNS-CNT SET was lower than in the GNS-C_60_ SET. Moreover, the charge stability diagrams for two devices were compared. The total area of Coulomb diamonds for the GNS-CNT device was smaller than for the device with the GNS-C_60_ island. Therefore, the zero-voltage region and the zero-conductance region of the GNS-CNT island were smaller than for the GNS-C_60_ island in their charge stability diagrams.

## Figures and Tables

**Table 1:** Important parameters extracted from Figure 12.

Diamond	Vds _min_ (V), Vds _max_ (V)	∆Vds (V)	Vg _min_ (V), Vg _max_ (V)	∆Vg (V)	Area of Diamonds (V^2^)	Total Areas (V^2^)
double GNS-CNT diamond 1	−0.465, 0.483	0.948	−0.958, −0.502	0.456	0.216	0.366
double GNS-CNT diamond 2	−0.493, −0.075	0.418	−0.313, −0.294	0.019	0.003	
double GNS-CNT diamond 3	−0.446, 0.465	0.911	0.056, 0.379	0.323	0.147	
double GNS- C_60_ diamond 1	−0.725, 0.744	1.469	−1.470, −0.749	0.721	0.529	1.372
double GNS- C_60_ diamond 2	−0.799, 0.818	1.617	−0.721, 0.066	0.787	0.636	
double GNS- C_60_ diamond 3	−0.446, 0.483	0.929	0.075, 0.521	0.446	0.207	

## Data Availability

This research does not report any data.
